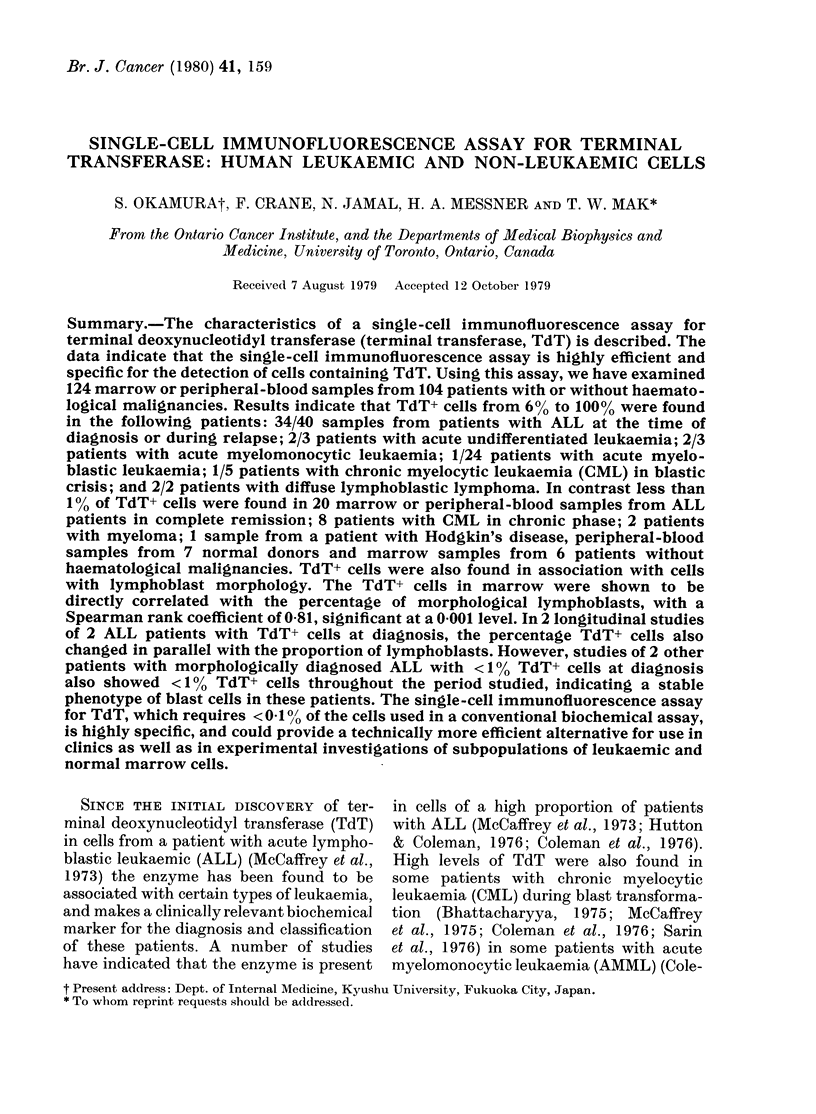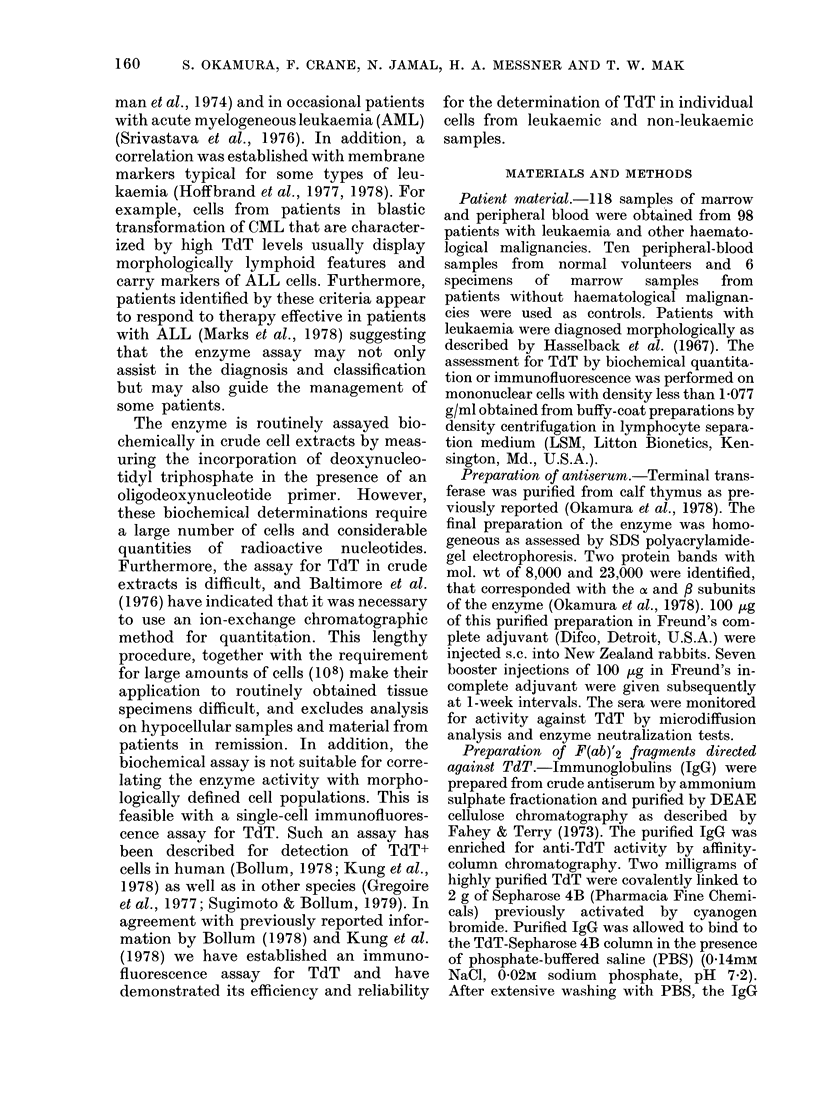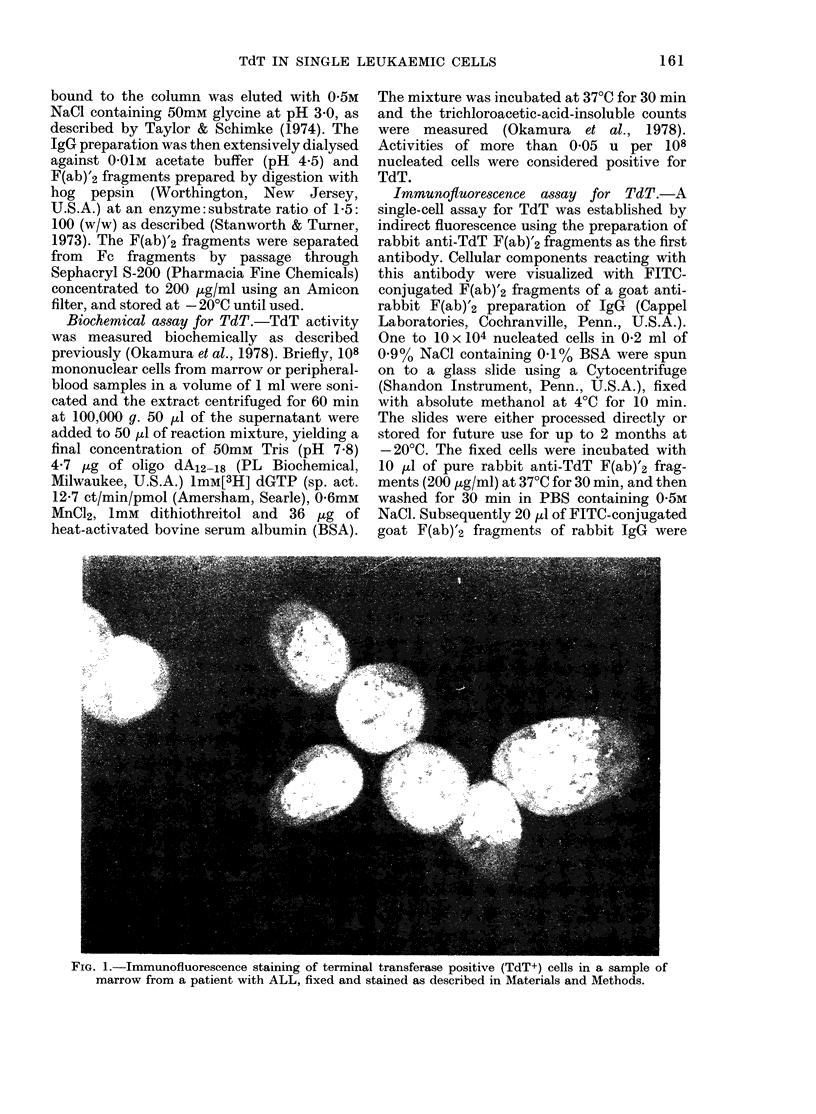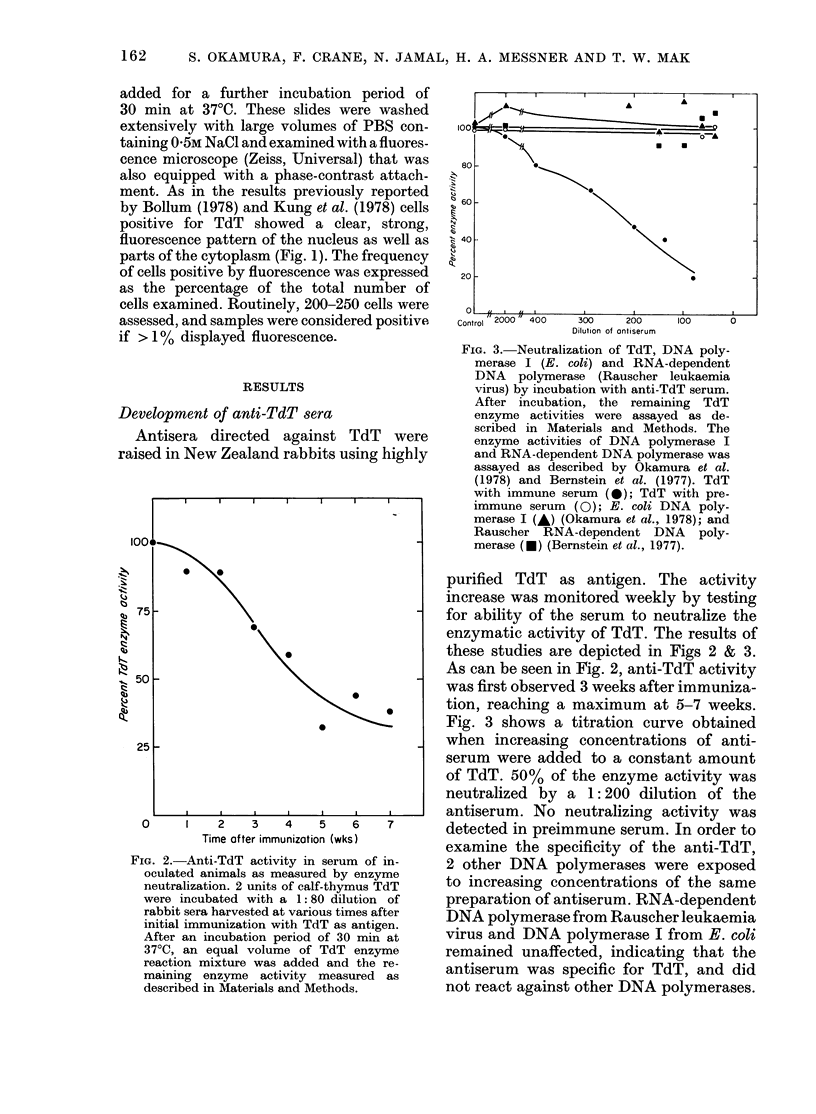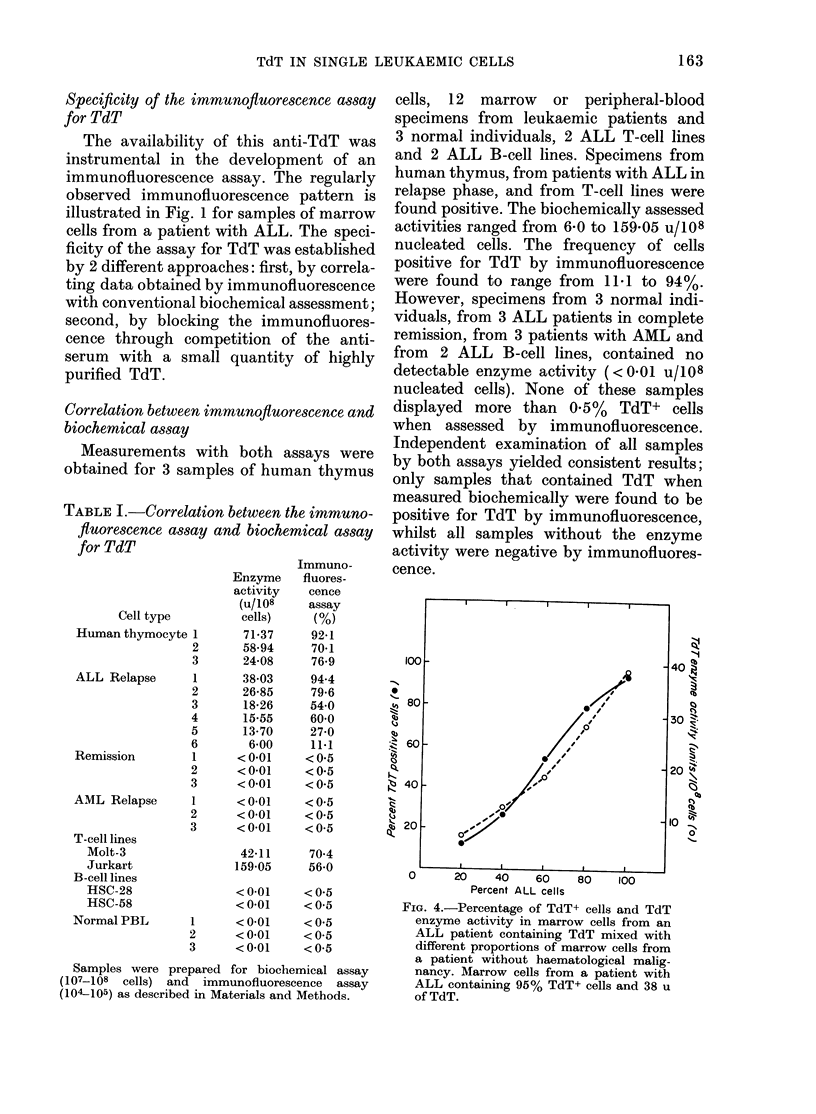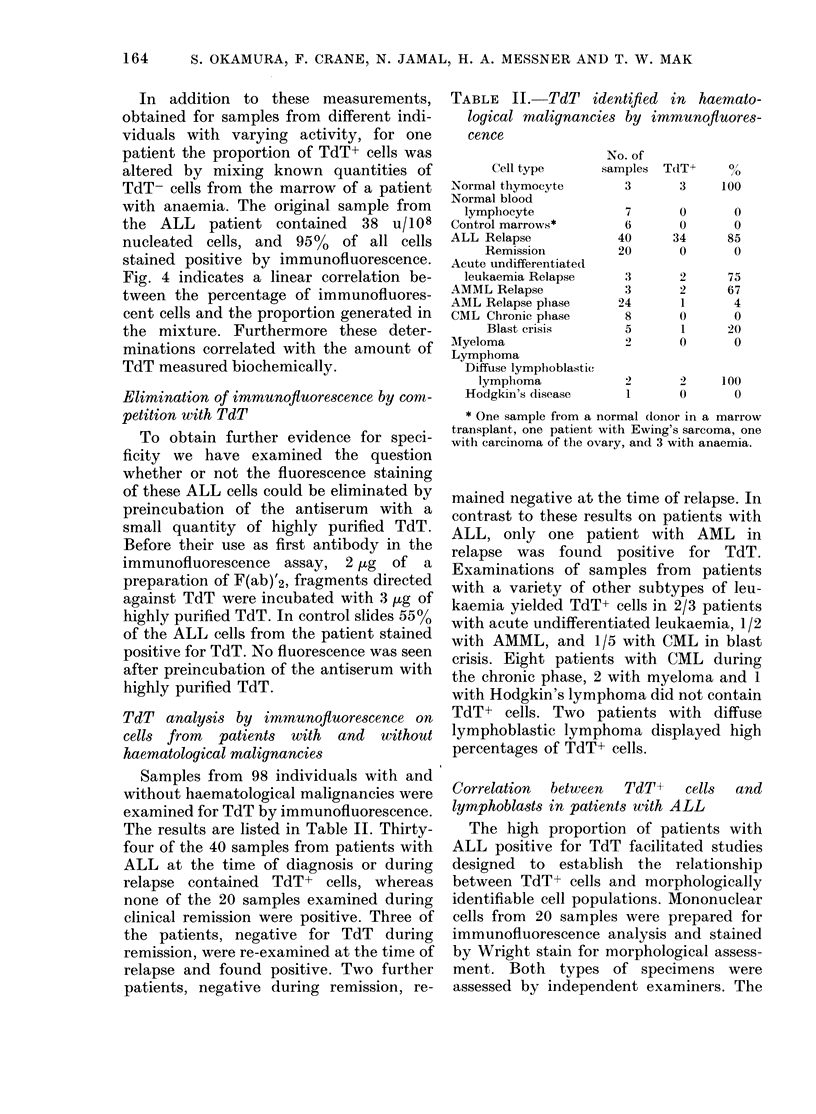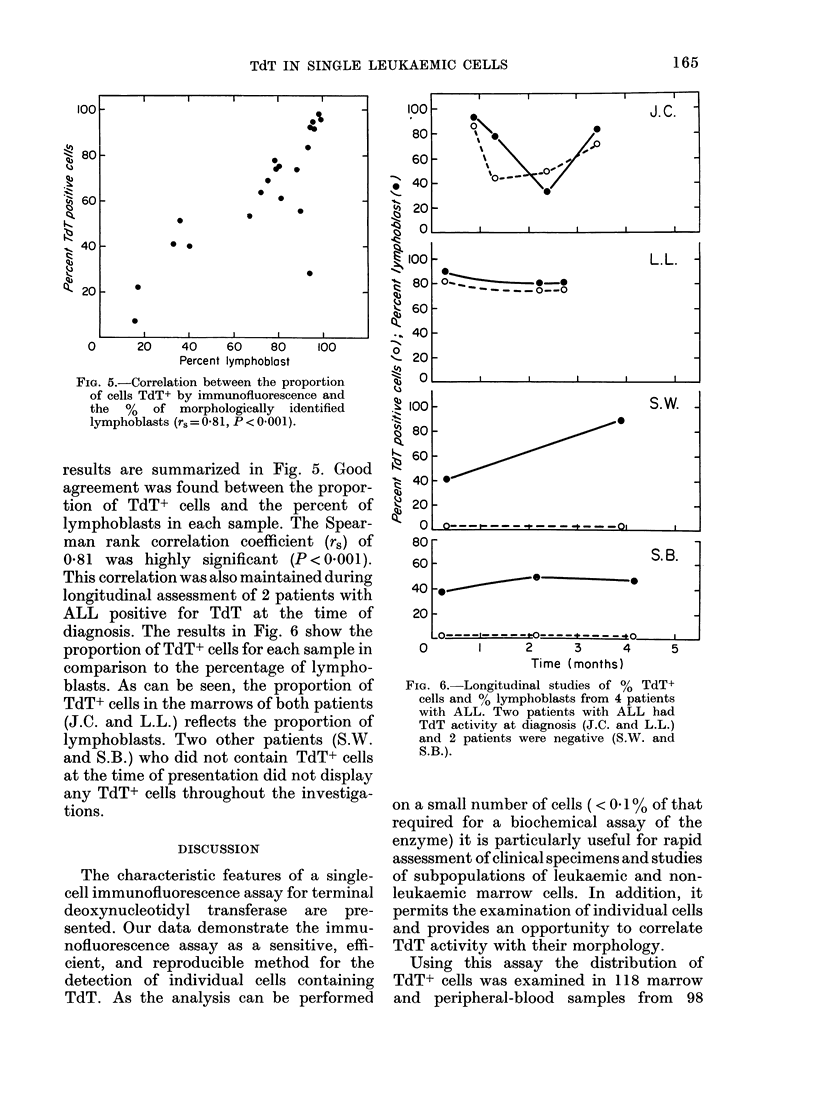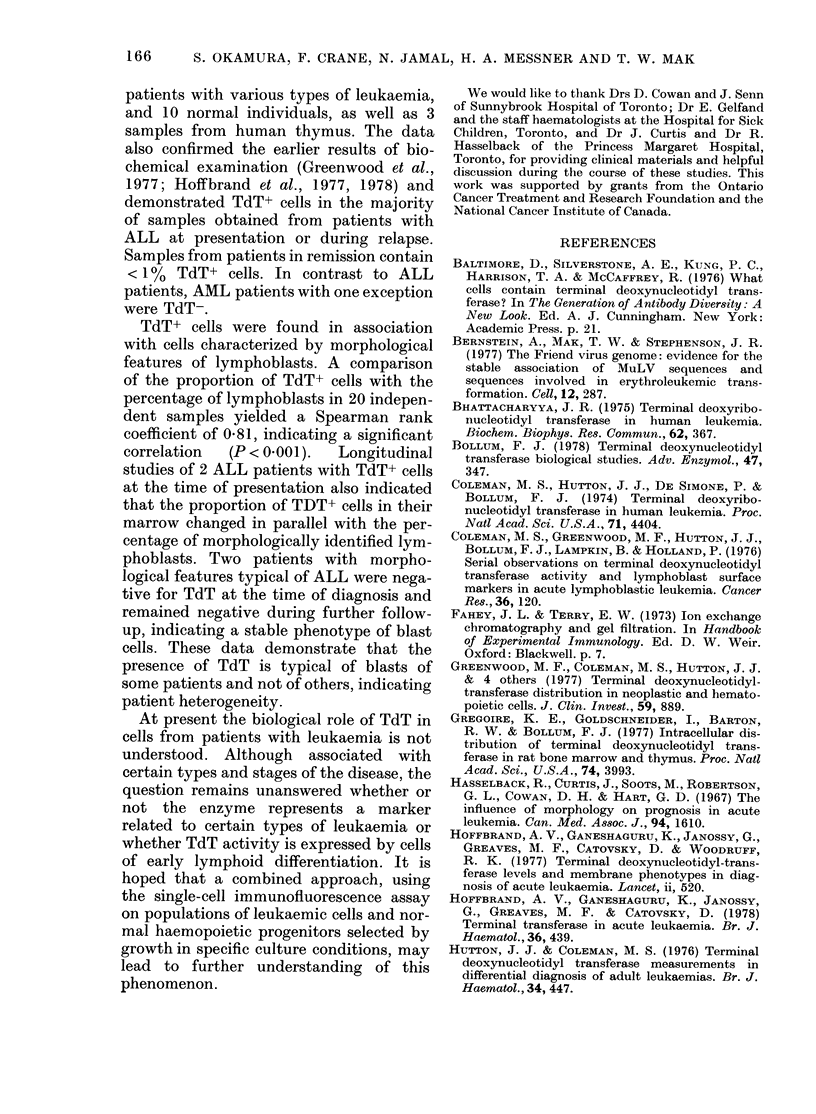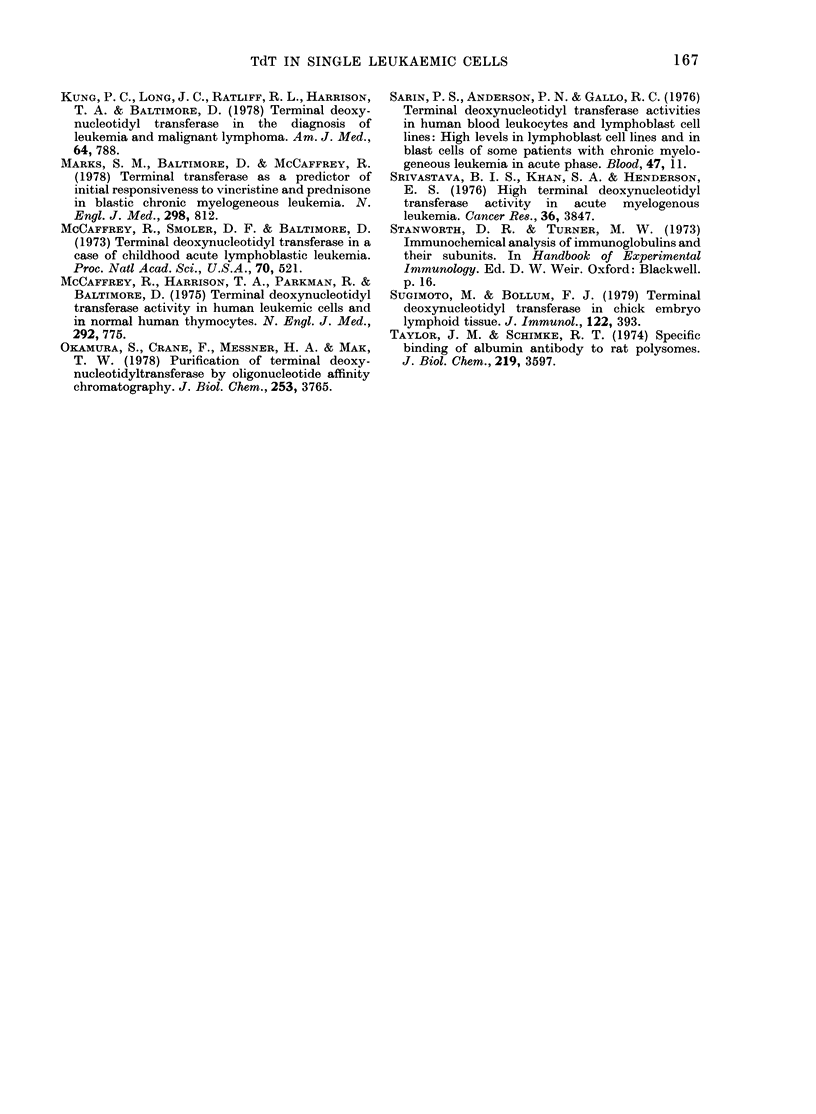# Single-cell immunofluorescence assay for terminal transferase: Human leukaemic and non-leukaemic cells

**DOI:** 10.1038/bjc.1980.26

**Published:** 1980-02

**Authors:** S. Okamura, F. Crane, N. Jamal, H. A. Messner, T. W. Mak

## Abstract

**Images:**


*Br. J. Cancer* (1980) **41**, 159

### SINGLE-CELL IMMUNOFLUORESCENCE ASSAY FOR TERMINAL TRANSFERASE: HUMAN LEUKAEMIC AND NON-LEUKAEMIC CELLS

S. OKAMURA†, F. CRANE, N. JAMAL, H. A. MESSNER AND T. W. MAK*

*From the Ontario Cancer Institute, and the Departments of Medical Biophysics and Medicine, University of Toronto, Ontario, Canada*

Received 7 August 1979 Accepted 12 October 1979

**Summary.—The characteristics of a single-cell immunofluorescence assay for terminal deoxynucleotidyl transferase (terminal transferase, TdT) is described. The data indicate that the single-cell immunofluorescence assay is highly efficient and specific for the detection of cells containing TdT. Using this assay, we have examined 124 marrow or peripheral-blood samples from 104 patients with or without haematological malignancies. Results indicate that TdT$^+$ cells from 6% to 100% were found in the following patients: 34/40 samples from patients with ALL at the time of diagnosis or during relapse; 2/3 patients with acute undifferentiated leukaemia; 2/3 patients with acute myelomonocytic leukaemia; 1/24 patients with acute myeloblastic leukaemia; 1/5 patients with chronic myelocytic leukaemia (CML) in blastic crisis; and 2/2 patients with diffuse lymphoblastic lymphoma. In contrast less than 1% of TdT$^+$ cells were found in 20 marrow or peripheral-blood samples from ALL patients in complete remission; 8 patients with CML in chronic phase; 2 patients with myeloma; 1 sample from a patient with Hodgkin's disease, peripheral-blood samples from 7 normal donors and marrow samples from 6 patients without haematological malignancies. TdT$^+$ cells were also found in association with cells with lymphoblast morphology. The TdT$^+$ cells in marrow were shown to be directly correlated with the percentage of morphological lymphoblasts, with a Spearman rank coefficient of 0·81, significant at a 0·001 level. In 2 longitudinal studies of 2 ALL patients with TdT$^+$ cells at diagnosis, the percentage TdT$^+$ cells also changed in parallel with the proportion of lymphoblasts. However, studies of 2 other patients with morphologically diagnosed ALL with <1% TdT$^+$ cells at diagnosis also showed <1% TdT$^+$ cells throughout the period studied, indicating a stable phenotype of blast cells in these patients. The single-cell immunofluorescence assay for TdT, which requires <0·1% of the cells used in a conventional biochemical assay, is highly specific, and could provide a technically more efficient alternative for use in clinics as well as in experimental investigations of subpopulations of leukaemic and normal marrow cells.**

SINCE THE INITIAL DISCOVERY of terminal deoxynucleotidyl transferase (TdT) in cells from a patient with acute lymphoblastic leukaemic (ALL) (McCaffrey *et al.*, 1973) the enzyme has been found to be associated with certain types of leukaemia, and makes a clinically relevant biochemical marker for the diagnosis and classification of these patients. A number of studies have indicated that the enzyme is present in cells of a high proportion of patients with ALL (McCaffrey *et al.*, 1973; Hutton & Coleman, 1976; Coleman *et al.*, 1976). High levels of TdT were also found in some patients with chronic myelocytic leukaemia (CML) during blast transformation (Bhattacharyya, 1975; McCaffrey *et al.*, 1975; Coleman *et al.*, 1976; Sarin *et al.*, 1976) in some patients with acute myelomonocytic leukaemia (AMML) (Cole-

† Present address: Dept. of Internal Medicine, Kyushu University, Fukuoka City, Japan.
* To whom reprint requests should be addressed.

man *et al.*, 1974) and in occasional patients with acute myelogeneous leukaemia (AML) (Srivastava *et al.*, 1976). In addition, a correlation was established with membrane markers typical for some types of leukaemia (Hoffbrand *et al.*, 1977, 1978). For example, cells from patients in blastic transformation of CML that are characterized by high TdT levels usually display morphologically lymphoid features and carry markers of ALL cells. Furthermore, patients identified by these criteria appear to respond to therapy effective in patients with ALL (Marks *et al.*, 1978) suggesting that the enzyme assay may not only assist in the diagnosis and classification but may also guide the management of some patients.

The enzyme is routinely assayed biochemically in crude cell extracts by measuring the incorporation of deoxynucleotidyl triphosphate in the presence of an oligodeoxynucleotide primer. However, these biochemical determinations require a large number of cells and considerable quantities of radioactive nucleotides. Furthermore, the assay for TdT in crude extracts is difficult, and Baltimore *et al.* (1976) have indicated that it was necessary to use an ion-exchange chromatographic method for quantitation. This lengthy procedure, together with the requirement for large amounts of cells ($10^8$) make their application to routinely obtained tissue specimens difficult, and excludes analysis on hypocellular samples and material from patients in remission. In addition, the biochemical assay is not suitable for correlating the enzyme activity with morphologically defined cell populations. This is feasible with a single-cell immunofluorescence assay for TdT. Such an assay has been described for detection of $TdT^+$ cells in human (Bollum, 1978; Kung *et al.*, 1978) as well as in other species (Gregoire *et al.*, 1977; Sugimoto & Bollum, 1979). In agreement with previously reported information by Bollum (1978) and Kung *et al.* (1978) we have established an immunofluorescence assay for TdT and have demonstrated its efficiency and reliability for the determination of TdT in individual cells from leukaemic and non-leukaemic samples.

### MATERIALS AND METHODS

*Patient material.*—118 samples of marrow and peripheral blood were obtained from 98 patients with leukaemia and other haematological malignancies. Ten peripheral-blood samples from normal volunteers and 6 specimens of marrow samples from patients without haematological malignancies were used as controls. Patients with leukaemia were diagnosed morphologically as described by Hasselback *et al.* (1967). The assessment for TdT by biochemical quantitation or immunofluorescence was performed on mononuclear cells with density less than 1·077 g/ml obtained from buffy-coat preparations by density centrifugation in lymphocyte separation medium (LSM, Litton Bionetics, Kensington, Md., U.S.A.).

*Preparation of antiserum.*—Terminal transferase was purified from calf thymus as previously reported (Okamura *et al.*, 1978). The final preparation of the enzyme was homogeneous as assessed by SDS polyacrylamide-gel electrophoresis. Two protein bands with mol. wt of 8,000 and 23,000 were identified, that corresponded with the $\alpha$ and $\beta$ subunits of the enzyme (Okamura *et al.*, 1978). 100 $\mu$g of this purified preparation in Freund's complete adjuvant (Difco, Detroit, U.S.A.) were injected s.c. into New Zealand rabbits. Seven booster injections of 100 $\mu$g in Freund's incomplete adjuvant were given subsequently at 1-week intervals. The sera were monitored for activity against TdT by microdiffusion analysis and enzyme neutralization tests.

*Preparation of $F(ab)'_2$ fragments directed against TdT.*—Immunoglobulins (IgG) were prepared from crude antiserum by ammonium sulphate fractionation and purified by DEAE cellulose chromatography as described by Fahey & Terry (1973). The purified IgG was enriched for anti-TdT activity by affinity-column chromatography. Two milligrams of highly purified TdT were covalently linked to 2 g of Sepharose 4B (Pharmacia Fine Chemicals) previously activated by cyanogen bromide. Purified IgG was allowed to bind to the TdT-Sepharose 4B column in the presence of phosphate-buffered saline (PBS) (0·14mM NaCl, 0·02M sodium phosphate, pH 7·2). After extensive washing with PBS, the IgG

bound to the column was eluted with 0·5M NaCl containing 50mM glycine at pH 3·0, as described by Taylor & Schimke (1974). The IgG preparation was then extensively dialysed against 0·01M acetate buffer (pH 4·5) and $F(ab)'_2$ fragments prepared by digestion with hog pepsin (Worthington, New Jersey, U.S.A.) at an enzyme:substrate ratio of 1·5: 100 (w/w) as described (Stanworth & Turner, 1973). The $F(ab)'_2$ fragments were separated from Fc fragments by passage through Sephacryl S-200 (Pharmacia Fine Chemicals) concentrated to 200 $\mu$g/ml using an Amicon filter, and stored at −20°C until used.

*Biochemical assay for TdT.*—TdT activity was measured biochemically as described previously (Okamura *et al.*, 1978). Briefly, $10^8$ mononuclear cells from marrow or peripheral-blood samples in a volume of 1 ml were sonicated and the extract centrifuged for 60 min at 100,000 *g*. 50 $\mu$l of the supernatant were added to 50 $\mu$l of reaction mixture, yielding a final concentration of 50mM Tris (pH 7·8) 4·7 $\mu$g of oligo $dA_{12-18}$ (PL Biochemical, Milwaukee, U.S.A.) 1mM[$^3$H] dGTP (sp. act. 12·7 ct/min/pmol (Amersham, Searle), 0·6mM $MnCl_2$, 1mM dithiothreitol and 36 $\mu$g of heat-activated bovine serum albumin (BSA). The mixture was incubated at 37°C for 30 min and the trichloroacetic-acid-insoluble counts were measured (Okamura *et al.*, 1978). Activities of more than 0·05 u per $10^8$ nucleated cells were considered positive for TdT.

*Immunofluorescence assay for TdT.*—A single-cell assay for TdT was established by indirect fluorescence using the preparation of rabbit anti-TdT $F(ab)'_2$ fragments as the first antibody. Cellular components reacting with this antibody were visualized with FITC-conjugated $F(ab)'_2$ fragments of a goat anti-rabbit $F(ab)'_2$ preparation of IgG (Cappel Laboratories, Cochranville, Penn., U.S.A.). One to $10 \times 10^4$ nucleated cells in 0·2 ml of 0·9% NaCl containing 0·1% BSA were spun on to a glass slide using a Cytocentrifuge (Shandon Instrument, Penn., U.S.A.), fixed with absolute methanol at 4°C for 10 min. The slides were either processed directly or stored for future use for up to 2 months at −20°C. The fixed cells were incubated with 10 $\mu$l of pure rabbit anti-TdT $F(ab)'_2$ fragments (200 $\mu$g/ml) at 37°C for 30 min, and then washed for 30 min in PBS containing 0·5M NaCl. Subsequently 20 $\mu$l of FITC-conjugated goat $F(ab)'_2$ fragments of rabbit IgG were

FIG. 1.—Immunofluorescence staining of terminal transferase positive ($TdT^+$) cells in a sample of marrow from a patient with ALL, fixed and stained as described in Materials and Methods.

added for a further incubation period of 30 min at 37°C. These slides were washed extensively with large volumes of PBS containing 0·5M NaCl and examined with a fluorescence microscope (Zeiss, Universal) that was also equipped with a phase-contrast attachment. As in the results previously reported by Bollum (1978) and Kung *et al.* (1978) cells positive for TdT showed a clear, strong, fluorescence pattern of the nucleus as well as parts of the cytoplasm (Fig. 1). The frequency of cells positive by fluorescence was expressed as the percentage of the total number of cells examined. Routinely, 200–250 cells were assessed, and samples were considered positive if >1% displayed fluorescence.

### RESULTS

### *Development of anti-TdT sera*

Antisera directed against TdT were raised in New Zealand rabbits using highly

FIG. 3.—Neutralization of TdT, DNA polymerase I (*E. coli*) and RNA-dependent DNA polymerase (Rauscher leukaemia virus) by incubation with anti-TdT serum. After incubation, the remaining TdT enzyme activities were assayed as described in Materials and Methods. The enzyme activities of DNA polymerase I and RNA-dependent DNA polymerase was assayed as described by Okamura *et al.* (1978) and Bernstein *et al.* (1977). TdT with immune serum (●); TdT with preimmune serum (○); *E. coli* DNA polymerase I (▲) (Okamura *et al.*, 1978); and Rauscher RNA-dependent DNA polymerase (■) (Bernstein *et al.*, 1977).

FIG. 2.—Anti-TdT activity in serum of inoculated animals as measured by enzyme neutralization. 2 units of calf-thymus TdT were incubated with a 1:80 dilution of rabbit sera harvested at various times after initial immunization with TdT as antigen. After an incubation period of 30 min at 37°C, an equal volume of TdT enzyme reaction mixture was added and the remaining enzyme activity measured as described in Materials and Methods.

purified TdT as antigen. The activity increase was monitored weekly by testing for ability of the serum to neutralize the enzymatic activity of TdT. The results of these studies are depicted in Figs 2 & 3. As can be seen in Fig. 2, anti-TdT activity was first observed 3 weeks after immunization, reaching a maximum at 5–7 weeks. Fig. 3 shows a titration curve obtained when increasing concentrations of antiserum were added to a constant amount of TdT. 50% of the enzyme activity was neutralized by a 1:200 dilution of the antiserum. No neutralizing activity was detected in preimmune serum. In order to examine the specificity of the anti-TdT, 2 other DNA polymerases were exposed to increasing concentrations of the same preparation of antiserum. RNA-dependent DNA polymerase from Rauscher leukaemia virus and DNA polymerase I from *E. coli* remained unaffected, indicating that the antiserum was specific for TdT, and did not react against other DNA polymerases.

*Specificity of the immunofluorescence assay for TdT*

The availability of this anti-TdT was instrumental in the development of an immunofluorescence assay. The regularly observed immunofluorescence pattern is illustrated in Fig. 1 for samples of marrow cells from a patient with ALL. The specificity of the assay for TdT was established by 2 different approaches: first, by correlating data obtained by immunofluorescence with conventional biochemical assessment; second, by blocking the immunofluorescence through competition of the antiserum with a small quantity of highly purified TdT.

*Correlation between immunofluorescence and biochemical assay*

Measurements with both assays were obtained for 3 samples of human thymus cells, 12 marrow or peripheral-blood specimens from leukaemic patients and 3 normal individuals, 2 ALL T-cell lines and 2 ALL B-cell lines. Specimens from human thymus, from patients with ALL in relapse phase, and from T-cell lines were found positive. The biochemically assessed activities ranged from 6·0 to 159·05 u/$10^8$ nucleated cells. The frequency of cells positive for TdT by immunofluorescence were found to range from 11·1 to 94%. However, specimens from 3 normal individuals, from 3 ALL patients in complete remission, from 3 patients with AML and from 2 ALL B-cell lines, contained no detectable enzyme activity (< 0·01 u/$10^8$ nucleated cells). None of these samples displayed more than 0·5% TdT+ cells when assessed by immunofluorescence. Independent examination of all samples by both assays yielded consistent results; only samples that contained TdT when measured biochemically were found to be positive for TdT by immunofluorescence, whilst all samples without the enzyme activity were negative by immunofluorescence.

TABLE I.—*Correlation between the immunofluorescence assay and biochemical assay for TdT*

| Cell type | | Enzyme activity (u/$10^8$ cells) | Immunofluorescence assay (%) |
|---|---|---|---|
| Human thymocyte | 1 | 71·37 | 92·1 |
| | 2 | 58·94 | 70·1 |
| | 3 | 24·08 | 76·9 |
| ALL Relapse | 1 | 38·03 | 94·4 |
| | 2 | 26·85 | 79·6 |
| | 3 | 18·26 | 54·0 |
| | 4 | 15·55 | 60·0 |
| | 5 | 13·70 | 27·0 |
| | 6 | 6·00 | 11·1 |
| Remission | 1 | < 0·01 | < 0·5 |
| | 2 | < 0·01 | < 0·5 |
| | 3 | < 0·01 | < 0·5 |
| AML Relapse | 1 | < 0·01 | < 0·5 |
| | 2 | < 0·01 | < 0·5 |
| | 3 | < 0·01 | < 0·5 |
| T-cell lines | | | |
| Molt-3 | | 42·11 | 70·4 |
| Jurkart | | 159·05 | 56·0 |
| B-cell lines | | | |
| HSC-28 | | < 0·01 | < 0·5 |
| HSC-58 | | < 0·01 | < 0·5 |
| Normal PBL | 1 | < 0·01 | < 0·5 |
| | 2 | < 0·01 | < 0·5 |
| | 3 | < 0·01 | < 0·5 |

Samples were prepared for biochemical assay ($10^7$–$10^8$ cells) and immunofluorescence assay ($10^4$–$10^5$) as described in Materials and Methods.

FIG. 4.—Percentage of TdT+ cells and TdT enzyme activity in marrow cells from an ALL patient containing TdT mixed with different proportions of marrow cells from a patient without haematological malignancy. Marrow cells from a patient with ALL containing 95% TdT+ cells and 38 u of TdT.

In addition to these measurements, obtained for samples from different individuals with varying activity, for one patient the proportion of TdT+ cells was altered by mixing known quantities of TdT− cells from the marrow of a patient with anaemia. The original sample from the ALL patient contained 38 u/$10^8$ nucleated cells, and 95% of all cells stained positive by immunofluorescence. Fig. 4 indicates a linear correlation between the percentage of immunofluorescent cells and the proportion generated in the mixture. Furthermore these determinations correlated with the amount of TdT measured biochemically.

*Elimination of immunofluorescence by competition with TdT*

To obtain further evidence for specificity we have examined the question whether or not the fluorescence staining of these ALL cells could be eliminated by preincubation of the antiserum with a small quantity of highly purified TdT. Before their use as first antibody in the immunofluorescence assay, 2 $\mu$g of a preparation of F(ab)'2, fragments directed against TdT were incubated with 3 $\mu$g of highly purified TdT. In control slides 55% of the ALL cells from the patient stained positive for TdT. No fluorescence was seen after preincubation of the antiserum with highly purified TdT.

*TdT analysis by immunofluorescence on cells from patients with and without haematological malignancies*

Samples from 98 individuals with and without haematological malignancies were examined for TdT by immunofluorescence. The results are listed in Table II. Thirty-four of the 40 samples from patients with ALL at the time of diagnosis or during relapse contained TdT+ cells, whereas none of the 20 samples examined during clinical remission were positive. Three of the patients, negative for TdT during remission, were re-examined at the time of relapse and found positive. Two further patients, negative during remission, remained negative at the time of relapse. In contrast to these results on patients with ALL, only one patient with AML in relapse was found positive for TdT. Examinations of samples from patients with a variety of other subtypes of leukaemia yielded TdT+ cells in 2/3 patients with acute undifferentiated leukaemia, 1/2 with AMML, and 1/5 with CML in blast crisis. Eight patients with CML during the chronic phase, 2 with myeloma and 1 with Hodgkin's lymphoma did not contain TdT+ cells. Two patients with diffuse lymphoblastic lymphoma displayed high percentages of TdT+ cells.

TABLE II.—*TdT identified in haematological malignancies by immunofluorescence*

| Cell type | No. of samples | TdT+ | % |
|---|---|---|---|
| Normal thymocyte | 3 | 3 | 100 |
| Normal blood lymphocyte | 7 | 0 | 0 |
| Control marrows* | 6 | 0 | 0 |
| ALL Relapse | 40 | 34 | 85 |
| Remission | 20 | 0 | 0 |
| Acute undifferentiated leukaemia Relapse | 3 | 2 | 75 |
| AMML Relapse | 3 | 2 | 67 |
| AML Relapse phase | 24 | 1 | 4 |
| CML Chronic phase | 8 | 0 | 0 |
| Blast crisis | 5 | 1 | 20 |
| Myeloma | 2 | 0 | 0 |
| Lymphoma | | | |
| Diffuse lymphoblastic lymphoma | 2 | 2 | 100 |
| Hodgkin's disease | 1 | 0 | 0 |

* One sample from a normal donor in a marrow transplant, one patient with Ewing's sarcoma, one with carcinoma of the ovary, and 3 with anaemia.

*Correlation between TdT+ cells and lymphoblasts in patients with ALL*

The high proportion of patients with ALL positive for TdT facilitated studies designed to establish the relationship between TdT+ cells and morphologically identifiable cell populations. Mononuclear cells from 20 samples were prepared for immunofluorescence analysis and stained by Wright stain for morphological assessment. Both types of specimens were assessed by independent examiners. The

Fig. 5.—Correlation between the proportion of cells TdT+ by immunofluorescence and the % of morphologically identified lymphoblasts ($r_S = 0{\cdot}81$, $P < 0{\cdot}001$).

results are summarized in Fig. 5. Good agreement was found between the proportion of TdT+ cells and the percent of lymphoblasts in each sample. The Spearman rank correlation coefficient ($r_S$) of 0·81 was highly significant ($P < 0{\cdot}001$). This correlation was also maintained during longitudinal assessment of 2 patients with ALL positive for TdT at the time of diagnosis. The results in Fig. 6 show the proportion of TdT+ cells for each sample in comparison to the percentage of lymphoblasts. As can be seen, the proportion of TdT+ cells in the marrows of both patients (J.C. and L.L.) reflects the proportion of lymphoblasts. Two other patients (S.W. and S.B.) who did not contain TdT+ cells at the time of presentation did not display any TdT+ cells throughout the investigations.

Fig. 6.—Longitudinal studies of % TdT+ cells and % lymphoblasts from 4 patients with ALL. Two patients with ALL had TdT activity at diagnosis (J.C. and L.L.) and 2 patients were negative (S.W. and S.B.).

### DISCUSSION

The characteristic features of a single-cell immunofluorescence assay for terminal deoxynucleotidyl transferase are presented. Our data demonstrate the immunofluorescence assay as a sensitive, efficient, and reproducible method for the detection of individual cells containing TdT. As the analysis can be performed on a small number of cells ( < 0·1% of that required for a biochemical assay of the enzyme) it is particularly useful for rapid assessment of clinical specimens and studies of subpopulations of leukaemic and non-leukaemic marrow cells. In addition, it permits the examination of individual cells and provides an opportunity to correlate TdT activity with their morphology.

Using this assay the distribution of TdT+ cells was examined in 118 marrow and peripheral-blood samples from 98

patients with various types of leukaemia, and 10 normal individuals, as well as 3 samples from human thymus. The data also confirmed the earlier results of biochemical examination (Greenwood *et al.*, 1977; Hoffbrand *et al.*, 1977, 1978) and demonstrated TdT+ cells in the majority of samples obtained from patients with ALL at presentation or during relapse. Samples from patients in remission contain $<1\%$ TdT+ cells. In contrast to ALL patients, AML patients with one exception were TdT−.

TdT+ cells were found in association with cells characterized by morphological features of lymphoblasts. A comparison of the proportion of TdT+ cells with the percentage of lymphoblasts in 20 independent samples yielded a Spearman rank coefficient of 0·81, indicating a significant correlation ($P < 0{\cdot}001$). Longitudinal studies of 2 ALL patients with TdT+ cells at the time of presentation also indicated that the proportion of TDT+ cells in their marrow changed in parallel with the percentage of morphologically identified lymphoblasts. Two patients with morphological features typical of ALL were negative for TdT at the time of diagnosis and remained negative during further follow-up, indicating a stable phenotype of blast cells. These data demonstrate that the presence of TdT is typical of blasts of some patients and not of others, indicating patient heterogeneity.

At present the biological role of TdT in cells from patients with leukaemia is not understood. Although associated with certain types and stages of the disease, the question remains unanswered whether or not the enzyme represents a marker related to certain types of leukaemia or whether TdT activity is expressed by cells of early lymphoid differentiation. It is hoped that a combined approach, using the single-cell immunofluorescence assay on populations of leukaemic cells and normal haemopoietic progenitors selected by growth in specific culture conditions, may lead to further understanding of this phenomenon.

We would like to thank Drs D. Cowan and J. Senn of Sunnybrook Hospital of Toronto; Dr E. Gelfand and the staff haematologists at the Hospital for Sick Children, Toronto, and Dr J. Curtis and Dr R. Hasselback of the Princess Margaret Hospital, Toronto, for providing clinical materials and helpful discussion during the course of these studies. This work was supported by grants from the Ontario Cancer Treatment and Research Foundation and the National Cancer Institute of Canada.

### REFERENCES

BALTIMORE, D., SILVERSTONE, A. E., KUNG, P. C., HARRISON, T. A. & McCAFFREY, R. (1976) What cells contain terminal deoxynucleotidyl transferase? In *The Generation of Antibody Diversity: A New Look*. Ed. A. J. Cunningham. New York: Academic Press. p. 21.

BERNSTEIN, A., MAK, T. W. & STEPHENSON, J. R. (1977) The Friend virus genome: evidence for the stable association of MuLV sequences and sequences involved in erythroleukemic transformation. *Cell*, **12**, 287.

BHATTACHARYYA, J. R. (1975) Terminal deoxyribonucleotidyl transferase in human leukemia. *Biochem. Biophys. Res. Commun.*, **62**, 367.

BOLLUM, F. J. (1978) Terminal deoxynucleotidyl transferase biological studies. *Adv. Enzymol.*, **47**, 347.

COLEMAN, M. S., HUTTON, J. J., DE SIMONE, P. & BOLLUM, F. J. (1974) Terminal deoxyribonucleotidyl transferase in human leukemia. *Proc. Natl Acad. Sci. U.S.A.*, **71**, 4404.

COLEMAN, M. S., GREENWOOD, M. F., HUTTON, J. J., BOLLUM, F. J., LAMPKIN, B. & HOLLAND, P. (1976) Serial observations on terminal deoxynucleotidyl transferase activity and lymphoblast surface markers in acute lymphoblastic leukemia. *Cancer Res.*, **36**, 120.

FAHEY, J. L. & TERRY, E. W. (1973) Ion exchange chromatography and gel filtration. In *Handbook of Experimental Immunology*. Ed. D. W. Weir. Oxford: Blackwell. p. 7.

GREENWOOD, M. F., COLEMAN, M. S., HUTTON, J. J. & 4 others (1977) Terminal deoxynucleotidyl-transferase distribution in neoplastic and hematopoietic cells. *J. Clin. Invest.*, **59**, 889.

GREGOIRE, K. E., GOLDSCHNEIDER, I., BARTON, R. W. & BOLLUM, F. J. (1977) Intracellular distribution of terminal deoxynucleotidyl transferase in rat bone marrow and thymus. *Proc. Natl Acad. Sci., U.S.A.*, **74**, 3993.

HASSELBACK, R., CURTIS, J., SOOTS, M., ROBERTSON, G. L., COWAN, D. H. & HART, G. D. (1967) The influence of morphology on prognosis in acute leukemia. *Can. Med. Assoc. J.*, **94**, 1610.

HOFFBRAND, A. V., GANESHAGURU, K., JANOSSY, G., GREAVES, M. F., CATOVSKY, D. & WOODRUFF, R. K. (1977) Terminal deoxynucleotidyl-transferase levels and membrane phenotypes in diagnosis of acute leukaemia. *Lancet*, ii, 520.

HOFFBRAND, A. V., GANESHAGURU, K., JANOSSY, G., GREAVES, M. F. & CATOVSKY, D. (1978) Terminal transferase in acute leukaemia. *Br. J. Haematol.*, **36**, 439.

HUTTON, J. J. & COLEMAN, M. S. (1976) Terminal deoxynucleotidyl transferase measurements in differential diagnosis of adult leukaemias. *Br. J. Haematol.*, **34**, 447.

KUNG, P. C., LONG, J. C., RATLIFF, R. L., HARRISON, T. A. & BALTIMORE, D. (1978) Terminal deoxynucleotidyl transferase in the diagnosis of leukemia and malignant lymphoma. *Am. J. Med.*, **64**, 788.

MARKS, S. M., BALTIMORE, D. & MCCAFFREY, R. (1978) Terminal transferase as a predictor of initial responsiveness to vincristine and prednisone in blastic chronic myelogeneous leukemia. *N. Engl. J. Med.*, **298**, 812.

MCCAFFREY, R., SMOLER, D. F. & BALTIMORE, D. (1973) Terminal deoxynucleotidyl transferase in a case of childhood acute lymphoblastic leukemia. *Proc. Natl Acad. Sci., U.S.A.*, **70**, 521.

MCCAFFREY, R., HARRISON, T. A., PARKMAN, R. & BALTIMORE, D. (1975) Terminal deoxynucleotidyl transferase activity in human leukemic cells and in normal human thymocytes. *N. Engl. J. Med.*, **292**, 775.

OKAMURA, S., CRANE, F., MESSNER, H. A. & MAK, T. W. (1978) Purification of terminal deoxynucleotidyltransferase by oligonucleotide affinity chromatography. *J. Biol. Chem.*, **253**, 3765.

SARIN, P. S., ANDERSON, P. N. & GALLO, R. C. (1976) Terminal deoxynucleotidyl transferase activities in human blood leukocytes and lymphoblast cell lines: High levels in lymphoblast cell lines and in blast cells of some patients with chronic myelogeneous leukemia in acute phase. *Blood*, **47**, 11.

SRIVASTAVA, B. I. S., KHAN, S. A. & HENDERSON, E. S. (1976) High terminal deoxynucleotidyl transferase activity in acute myelogenous leukemia. *Cancer Res.*, **36**, 3847.

STANWORTH, D. R. & TURNER, M. W. (1973) Immunochemical analysis of immunoglobulins and their subunits. In *Handbook of Experimental Immunology*. Ed. D. W. Weir. Oxford: Blackwell. p. 16.

SUGIMOTO, M. & BOLLUM, F. J. (1979) Terminal deoxynucleotidyl transferase in chick embryo lymphoid tissue. *J. Immunol.*, **122**, 393.

TAYLOR, J. M. & SCHIMKE, R. T. (1974) Specific binding of albumin antibody to rat polysomes. *J. Biol. Chem.*, **219**, 3597.